# Solitary Extramedullary Plasmacytoma of the Larynx and Secondary Laryngeal Involvement in Plasma Cell Myeloma: Single-Centre Retrospective Analysis and Systematic Literature Review

**DOI:** 10.3390/jcm11154390

**Published:** 2022-07-28

**Authors:** Elżbieta Szczepanek, Joanna Drozd-Sokołowska, Jacek Sokołowski, Anna Rzepakowska, Arkadiusz Moskwa, Jakub Pachla, Jakub Grzybowski, Katarzyna Woźnica, Kazimierz Niemczyk, Krzysztof Jamroziak

**Affiliations:** 1 Department of Otorhinolaryngology, Head and Neck Surgery, Medical University of Warsaw, 02-097 Warsaw, Poland; jacsokolowski@gmail.com (J.S.); arzepakowska@wum.edu.pl (A.R.); amoskwa2001@gmail.com (A.M.); santiago5@tlen.pl (J.P.); kniemczyk@wum.edu.pl (K.N.); 2 Doctoral School in Medical Sciences and Health Sciences, Jagiellonian University Medical College, 31-530 Cracow, Poland; 3 Department of Hematology, Transplantation and Internal Medicine, Medical University of Warsaw, 02-091 Warsaw, Poland; johna.dr@poczta.fm (J.D.-S.); k.m.jamroziak@gmail.com (K.J.); 4 Department of Pathology, Medical University of Warsaw, 02-091 Warsaw, Poland; jgrzybowski@wum.edu.pl; 5 Faculty of Mathematics and Information Science, Warsaw University of Technology, 00-661 Warsaw, Poland; k.woznica@mini.pw.edu.pl

**Keywords:** extramedullary plasmacytoma, plasma cell myeloma, multiple myeloma, larynx

## Abstract

The involvement of the larynx in plasma cell myeloma (PCM) may manifest as solitary extramedullary plasmacytoma of the larynx (sEMP-L) or as infiltration of the larynx during newly diagnosed or relapsed systemic disease with bone marrow involvement (plasma cell myeloma with laryngeal involvement, PCM-L). To increase knowledge about these rare conditions, we performed a retrospective analysis along with a comprehensive literature review of cases of sEMP-L or PCM-L. Six patients (two sEMP-L and four PCM-L) were identified in our tertiary laryngological centre from 2009 to 2021, constituting 0.88% of all malignant laryngeal tumours. The literature search yielded 187 cases, including 152 sEMP-L and 35 sPCM-L. A comparison of baseline characteristics between sEMP-L and PCM-L performed in the combined cohort of cases from literature review and retrospective analysis revealed that patients with sEMP-L were younger (56 vs. 64 years, *p* ≤ 0.001) and presented less commonly with thyroid or cricoid cartilage involvement (2.2% vs. 30.8%, *p* ≤ 0.001). The prognosis of sEMP-L was better than PCM-L (overall survival 86% vs. 55% at 5 years, *p* = 0.002). Analysis of potential factors that could influence progression-free survival (PFS) in the group of sEMP-L revealed that male sex and cartilage involvement negatively affected PFS in univariate analyses, while only cartilage involvement retained statistical significance in multivariate analysis (HR = 19.94, *p* = 0.024). In conclusion, PCM with laryngeal involvement is sporadic. Secondary involvement of the larynx during PCM might be more common than sEMP-L and is associated with worse survival. The involvement of cartilage adversely influences the outcome of sEMP-L.

## 1. Introduction

Plasma cell myeloma (PCM) belongs to mature B-cell lymphoid neoplasm and is typically characterized by the infiltration of the bone marrow by clonal plasma cells and the presence of a monoclonal protein in serum and/or in urine [1]. Under some circumstances, the disease may present as a single extraosseous lesion, called either solitary extraosseous or extramedullary plasmacytoma [2]. Extramedullary plasmacytomas constitute solely 3–5% of all plasma cell dyscrasias, with 80% occurring in the upper respiratory tract, including the oropharynx, nasopharynx, sinuses, and the larynx [2]. Despite this significant relative frequency, the amount of detailed epidemiological data debating the involvement of specific structures within the upper respiratory tract, such as the larynx, is scarce [3,4].

While squamous cell carcinoma is the most common primary malignancy of the larynx, with an annual incidence of 2.76 cases/year per 100,000 inhabitants [5], it is of utmost importance to take cognizance of non-epithelial neoplasms, including malignancies of lymphoid origin such as PCM, in the differential diagnosis of new larynx lesions. In the case of such single lesion, i.e., solitary extramedullary plasmacytoma of the larynx (sEMP-L), the treatment of choice is usually local intervention with surgery or radiotherapy or a combination of these methods [3]. However, laryngeal involvement by PCM may also occur in the course of a systemic disease when typical bone marrow involvement is accompanied by extramedullary infiltration of the larynx by neoplastic plasma cells. In such a clinical situation, the larynx can either be the sole site of extramedullary involvement or just one of many extramedullary lesions. There is even less available epidemiological data regarding clinical scenarios in which laryngeal involvement in systemic PCM (PCM-L) is confirmed than in the case of sEMP-L [6,7]. Furthermore, there are no specific recommendations on the staging of the laryngeal involvement by PCM, as well as no dedicated recommendations to guide diagnostic workup and support treatment decisions.

Therefore, we launched this retrospective study to elucidate the issue of the incidence of laryngeal involvement by PCM, both solitary and in the course of bone marrow disease. We utilized a large cohort of consecutive patients with malignant neoplasms of the larynx diagnosed in a single tertiary laryngological academic centre over a relatively long period of time. Moreover, taking into consideration the rarity of the diagnosis, we also performed a literature search to summarize the world experience with PCM of the larynx, to provide patients’ and diseases’ characteristics and to analyze potential factors with influence on outcomes.

## 2. Materials and Methods

### 2.1. Study Design

This study comprises a single-centre retrospective analysis and a systematic literature review on the involvement of the larynx in PCM. In both study parts, the identified cases were divided into two categories depending on the type of laryngeal involvement, namely solitary extramedullary plasmacytoma of the larynx (sEMP-L) and laryngeal involvement during systemic disease with bone marrow involvement (PCM-L). Concerning the second category, two different clinical scenarios were possible including newly diagnosed systemic PCM with concomitant involvement of the larynx and relapsed systemic PCM with secondary involvement of the larynx as a single site of the disease or along with other sites including bone marrow and/or extramedullary lesions. The study was approved by the Ethical Committee of the Medical University of Warsaw (no. AKBE/22/2022).

### 2.2. Retrospective Analysis

A retrospective analysis of patients with laryngeal neoplasms diagnosed in the Department of Otorhinolaryngology Head and Neck Surgery of the Medical University of Warsaw was performed to identify all cases with diagnosis of isolated or secondary laryngeal involvement in PCM established between 2009 and 2021. The inclusion criterion was larynx infiltration by clonal plasma cells, which was confirmed by immunohistochemistry of the laryngeal specimen.

### 2.3. Systematic Review

A systematic PubMed/Medline review on laryngeal involvement in PCM was performed for articles published until April 2022 in the English language with the following medical searching headings (Title/Abstract/MesH word)—larynx and plasmacytoma (or plasmocytoma); larynx and multiple myeloma; larynx and EMP; laryngeal and (or plasmocytoma); laryngeal and plasmacytoma; laryngeal and multiple myeloma; laryngeal and EMP. Full articles of the eligible papers were retrieved. If the full text was unavailable in some papers, the abstracts were utilised to obtain all possible information. Several case reports were also obtained by cross-reference checking. Where available, data were collected about the patient’s demographics (age and sex) and features related to the disease, including clinical symptoms at presentation, localisation of the tumour, involvement of cartilage (thyroid or cricoid), mode of therapy, the dose of radiotherapy (if used), time of follow-up and outcomes.

### 2.4. Statistical Analysis

The statistical analysis was performed using the R software, version 4.2.0. The patients’ cohort included a combined group of patient cases identified through single-centre retrospective analysis and PubMed/Medline literature review. For specific analyses, the patients were divided into sEMP-L and PCM-L categories. In these two groups, demographics, disease-related features, and survival were compared. Categorical variables were tested for statistical significance using Fisher’s exact test. The continuous age variable was expressed as mean ± SEM (standard error of the mean) and range of values (minimum and maximum). A non-parametric Mann–Whitney test was used to compare the mean values of patients’ age. Follow-up was censored at the date of the last contact among survivors. Progression-free survival (PFS) was defined as the time from the diagnosis to relapse or progressive disease or death from any cause, whichever came first. Overall survival (OS) was calculated from the time of diagnosis to death from any cause. Survival curves were plotted using Kaplan–Meier method. Furthermore, in the group of patients with sEMP-L (and not in the small and heterogenous PCM-L group), the impact of several demographic and disease-related variables on PFS was tested. To assess the impact of a single variable on the progression in the group of patients with sEMP-L, the log-rank test and survival curves are used. Next, to take into consideration many variables influencing progression in multivariate survival analyses, Cox regression was applied. In the multivariate Cox model, variables determined in backward feature selection (sex, cartilage involvement, type of therapy) were included. In that model, the hazard ratio was calculated, and 95% confidence intervals (CI) were determined. A *p*-value of <0.05 was considered significant.

## 3. Results

### 3.1. Results of Retrospective Analysis

This retrospective analysis was performed in the Department of Otorhinolaryngology, Head and Neck Surgery of the Medical University of Warsaw, Poland, an important tertiary laryngology centre for an urban agglomeration of 1.78 million citizens and a territorial unit with an additional 3.64 million inhabitants. From 2009 to 2021, we identified 659 patients diagnosed with epithelial laryngeal neoplasms and 22 with nonepithelial laryngeal neoplasms diagnosed in the Department. Within the group of nonepithelial larynx neoplasms, there were six patients with involvement of the larynx by PCM, including two cases of sEMP-L and four cases of PMC-L. Nonepithelial laryngeal neoplasms accounted for 3.23% of all malignant tumours of the larynx while cases with PCM involvement accounted for 0.88% of malignant laryngeal tumours. The basic demographic data, tumour localisation, treatment, and outcomes for the six patients with PCM are summarised in Table 1. Some additional details of the diagnostic process, the clinical picture and the course of the disease, are given below.

Two of the six patients (Patients 1 and 2 from Table 1) were diagnosed with sEMP-L. The first patient, a 70-year-old man with a history of smoking and asbestos exposure, underwent a total of six laryngeal directoscopies due to chronic cough, hoarseness, mild dyspnoea, and recurrent hypertrophic lesions within the larynx. Only the last biopsy from October 2019 provided a conclusive result with lambda light chains restricted infiltration of plasma cells. The patient was referred to the Department of Hematology, where as a result of the performed diagnostic tests (bone marrow aspiration, flow cytometry, bone marrow biopsy, electrophoresis and immunofixation of serum and urine, computed tomography (CT) body scan) sEMP-L was diagnosed. The patient was qualified for radical 3D-MRT (three-dimensional magnetic resonance tomosynthesis) radiotherapy to the larynx, which resulted in complete remission. At the last follow-up visit, 18 months post-treatment, no local recurrence or progression to the systemic form of PCM was found [8]. The second patient, a 59-year-old man, was referred to the Department of Otorhinolaryngology due to the polypoid mass localised on the epiglottis and right aryepiglottic fold (no symptoms were observed—accidental finding). The patient had no history of smoking. Directoscopy of the larynx was performed in February 2013, and histopathological examination revealed the infiltration of kappa light chains secreting plasma cells. Diagnostic tests confirmed sEMP-L. The radiotherapy was performed, and after 111 months of follow-up, no signs of systemic recurrence or progression were seen.

**Figure 1:**
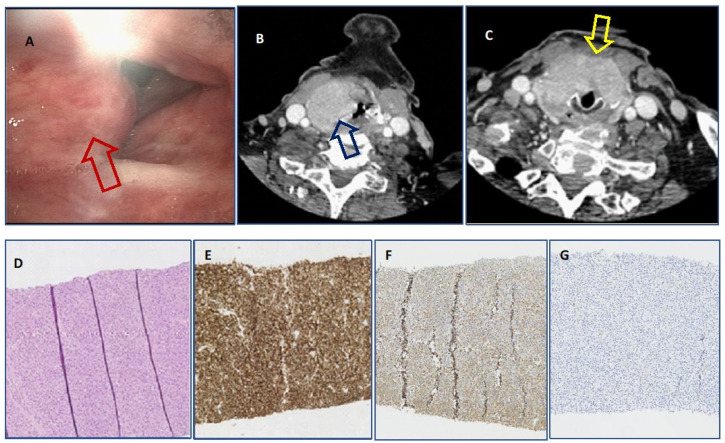
Endoscopic view of hypertrophic right ventricular fold (red arrow) of patient’s larynx infiltrated with extramedullary plasmacytoma (**A**). Neck CT scan of large tumour mass involving laryngeal structures on the right side with thyroid cartilage destruction and extralaryngeal infiltration of infrahyoid muscles (blue arrow) with increased tissue enhancement (**B**) and progressing to the subglottic level bilaterally with anterior part of cricoid cartilage destruction (yellow arrow) (**C**). Histological view of hematoxylin and eosin staining of core biopsy laryngeal specimen with mature plasma cells x10 (**D**). Immunohistochemical positivity for CD138 antigen x10 (**E**) with weak cytoplasmic kappa light chain positivity x10 (**F**) and complete absence of lambda light chain expresssion x10 (**G**).

Secondary laryngeal involvement in PCM (PCM-L) was diagnosed in four patients (Patients 3–6 from Table 1). The first patient, a 72-year-old female with a 3-year course of PCM, after several lines of chemotherapy, was admitted to the Department of Otorhinolaryngology due to dyspnea and hoarseness lasting for 2 months with a suspicion of a second primary malignancy (laryngeal cancer). The endoscopic view revealed a tumour of the right aryepiglottic fold, right piriform sinus, both vocal cords, and subglottic area. During the hospitalization, directoscopy with subsequent tracheotomy was performed. The PCM infiltration was diagnosed based on the laryngeal biopsy in November 2009. The patient was referred to the Department of Hematology, where systemic progression of PCM was diagnosed, with pancytopenia resulting from extensive bone marrow infiltration by clonal plasma cells. Due to the patient’s poor general condition and the PCM’s advanced stage, further causal treatment was not initiated. The patient died due to the PCM progression at 3.5 years from the diagnosis of PCM and 3 weeks after the diagnosis of laryngeal involvement. The next patient was an 84-year-old man with a 1.5-year course of PCM after treatment with six cycles of CTD (cyclophosphamide, thalidomide, dexamethasone) chemotherapy. The patient developed pain in the neck area (1.5 years after PCM diagnosis), which lasted for approximately 2 months. Endoscopic examination of the larynx revealed a proliferative lesion on the left vocal cord. Directoscopy of the larynx was performed in August 2014, and histopathological examination revealed PCM infiltration. Radiotherapy was performed, and no local recurrence was observed. The patient died 33 months after the diagnosis of PCM and 14 months after the diagnosis of laryngeal involvement due to systemic progression. Two other patients (men aged 78 and 89) who had not been previously diagnosed with PCM were diagnosed at the Department of Otorhinolaryngology with a clonal plasma cell infiltration of the larynx. A 78-year-old patient was admitted to the Otorhinolaryngology Department due to dyspnea in July 2021. The endoscopic examination of this patient showed a hypertrophic right ventricular fold. The histopathological examination revealed the infiltration of the larynx with the plasma cells. Moreover, there were disseminated neoplastic lesions within bones, including the jaw, the collarbone, and the sternum (Figure 1).

**Figure 2:**
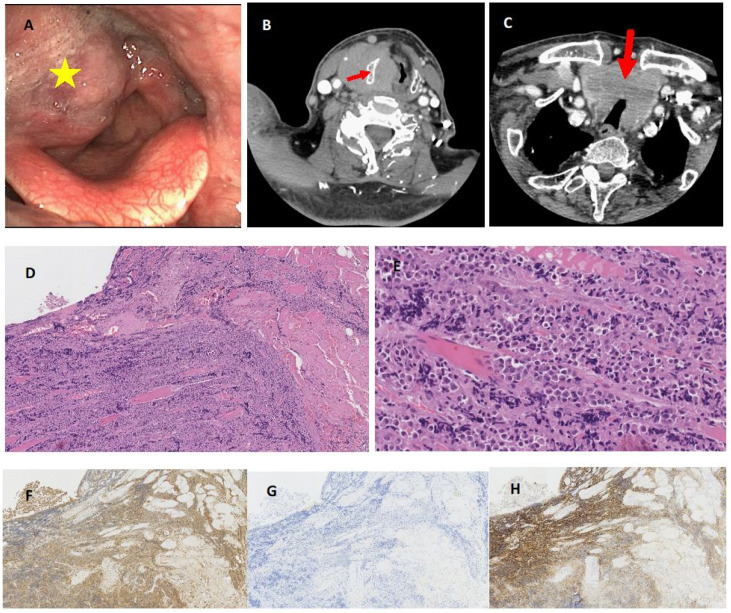
Endoscopic view of bulky extramedullary plasmacytoma invading right side of the larynx and hypopharynx (yellow asterisk) (**A**). Neck CT scan of large tumour mass involving laryngeal structures at the glottic and supraglottic level, with extralaryngeal spread and thyroid cartilage sclerotization and partial destruction (red arrow) without increased tissue enhancement (**B**). The infiltration involves also both thyroid gland lobes with moderate narrowing of the trachea (red arrow) (**C**). Histological view of hematoxylin and eosin staining of laryngeal specimen with plasma cells infiltrating muscles x10 (**D**) x40 (**E**) and strong cytoplasmic kappa light chain positivity x10 (**F**) with complete absence of lambda light chain expression x10 (**G**) and immunohistochemical positivity for CD138 antigen x10 (**H**).

The last patient, an 89-year-old male, was admitted to the Department of Otorhinolaryngology with dyspnea lasting for several weeks. The endoscopic examination of the larynx revealed a bulky tumour invading the right side of the larynx (right aryepiglottic fold, ventricular fold, vocal cord) and hypopharynx. Directoscopy and tracheostomy were performed in November 2021, and histopathological examination revealed lambda light chains secreting plasma cells suggesting extraosseous plasmacytoma (Figure 2).

Subsequently, based on further studies, both patients described above were diagnosed with systemic PCM with extramedullary lesions in the larynx. The patients were transferred to the Department of Hematology, where steroid pre-treatment was introduced. Due to poor general condition, comorbidities, and no sign of improvement after steroid therapy, both patients were not qualified for systemic chemotherapy. Despite the implemented symptomatic management, the patients died, respectively, 1.5 and 2 months after the diagnosis of PCM and PCM laryngeal involvement in the course of disease progression. These patients’ endoscopic views, CT scans, and histopathological pictures are presented in Figure 1 and Figure 2.

### 3.2. Literature Review

**Figure 3:**
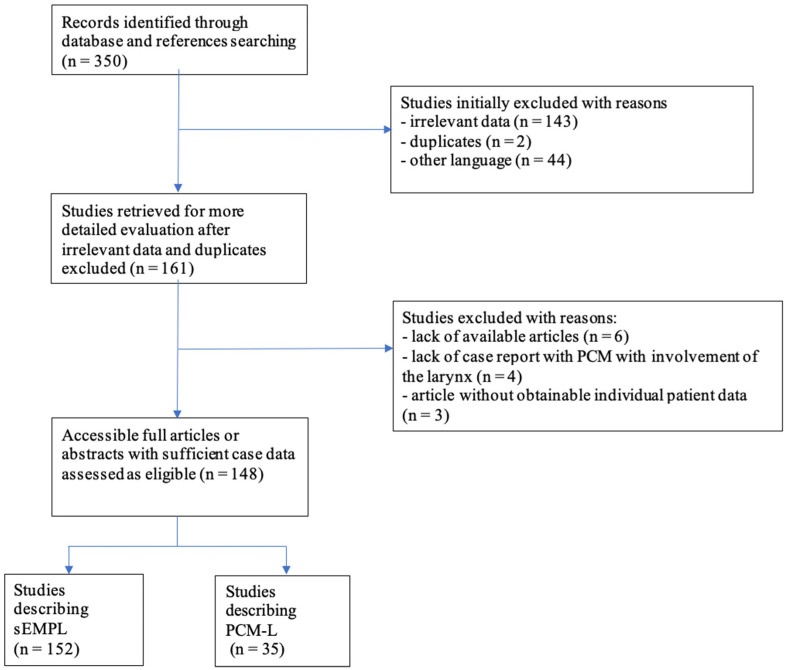
Graphical representation of the process of selecting articles for literature review regarding laryngeal involvement in plasma cell myeloma. sEMPL—solitary extramedullary 4myeloma with involvement of the bone marrow and secondary involvement of the larynx. PCM-L—secondary laryngeal involvement in plasma cell myeloma.

As a result of our literature search, 350 articles were identified, yielding a total of 148 articles reporting 187 cases of laryngeal involvement in PCM, including 152 cases of sEMP-L and 35 cases of PCM-L. The method of selecting articles for the review is presented in Figure 3.

Most of the identified articles were case reports or small case series. The demographic data, localisation of the tumour, treatment, and outcomes from the literature review are summarized in Table 2 for sEMP-L cases and in Table 3 regarding patients with PCM-L.

**Figure 4:**
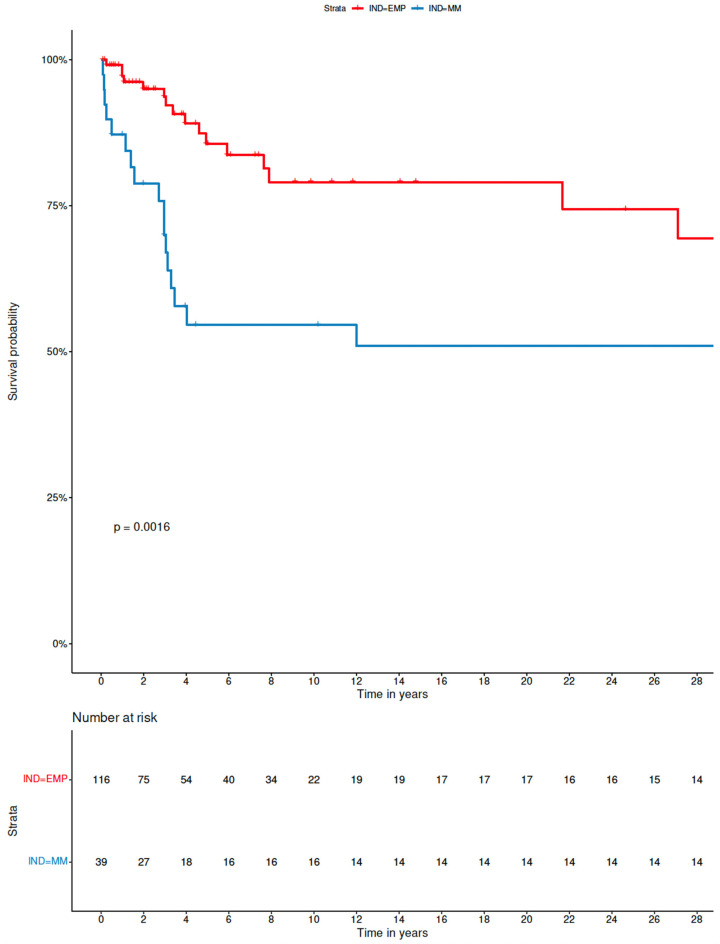
Comparison of overall survival by Kaplan–Meier method between patients with solitary extramedullary plasmacytoma of the larynx (sEMPL) and patients with plasma cell myeloma with involvement of the bone marrow and extramedullary infiltration of the larynx (PCM-L) in the combined cohort of patients from the literature review (*n* = 173) and retrospective analysis (*n* = 6). P derived from log-rank test.

Next, we compared the available demographic and clinical data between the groups of patients with sEMP-L and PCM-L in the combined cohort of patient cases identified through our single-centre retrospective analysis and PubMed/Medline literature review. Interestingly, we found some significant differences between both groups. The median age of patients from the sEMP-L group was 56.0 (range, 11–88) years, while patients with secondary involvement of the larynx (PCM-L) had a median age of 64.0 (range, 44–89) years (*p* < 0.001). Furthermore, there was a significant difference in sex proportion between both cohorts, with 33.6% of female patients in the sEMP-L group and only 17.9% of females among patients with PCM-L (*p* = 0.038). Moreover, patients with sEMP-L had supraglottis, glottis, or both localisations involved in 75.4% of cases, while in those with PCM-L, these localisations were involved in 48.7% (*p* = 0.0027). The cartilages of the larynx were not involved in 97.8% of patients with sEMP-L, but such involvement was relatively common (30.8%) in patients with PCM-L (*p* < 0.001). Finally, we compared the OS between patients with sEMP-L and PCM-L. As expected, the patients with PCM-L had less favourable prognosis than cases with sEMP-L with 5-year OS of 55% (95% CI, 40–74%) vs. 86% (95% CI, 79–94%) and 10-year of 55% (95% CI, 40–74%) vs. 81% (95% CI, 72–90%), respectively (Figure 4).

Due to the heterogeneity of PCM-L group that included cases with larynx involvement at diagnosis or at relapse and a variety of modes of treatment used, a comparison of PFS was not performed for this population.

### 3.3. Analysis of Factors Influencing Outcome of sEMP-L

Subsequently, we assessed the significance of potential demographic and clinical factors that could impact the duration of PFS in the group of patients with sEMP-L. This cohort included 2 cases identified in our retrospective analysis and 152 cases found through the literature review, although the survival data were available only for 120 patients.

**Figure 5:**
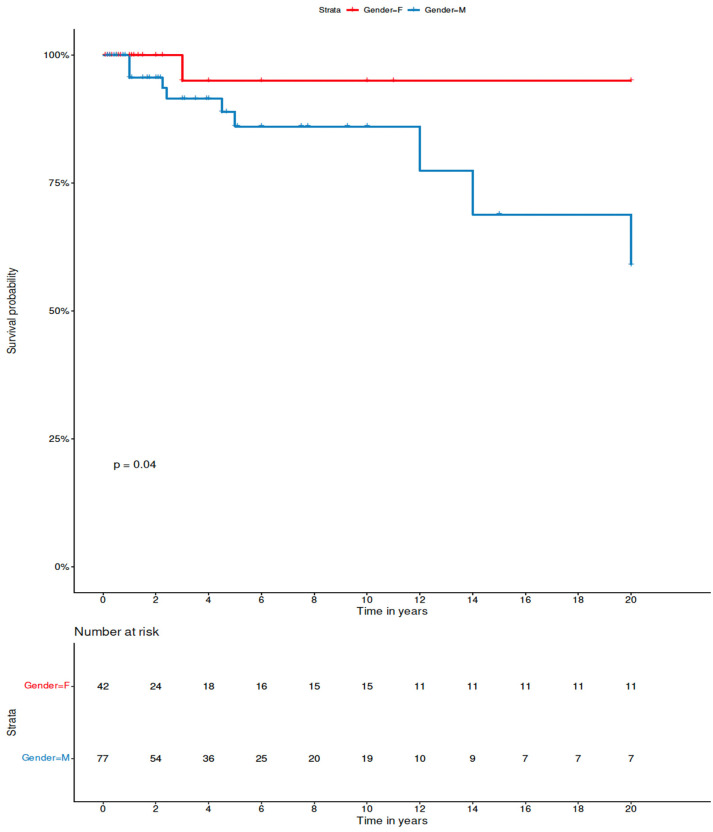
Comparison of progression-free survival by Kaplan–Meier method between male and female patients with solitary extramedullary plasmacytoma of the larynx in the combined cohort of patients from the literature review and retrospective analysis. P derived from log-rank test.

Among variables tested, only the sex and thyroid cartilage involvement did impact on the outcome, with the male sex and the presence of cartilage involvement being associated with shorter PFS in univariate analysis (the Kaplan–Meier curve illustrating the negative influence of the male sex on the probability of PFS is presented in Figure 5). Interestingly, analysis of potential impact of the dose of radiotherapy dichotomized as ≤4000 cGy and >4000 cGy did not show significant differences in regard to PFS probability (*p* = 0.51).

In multivariate analysis, only the cartilage involvement retained its statistical significance, with an HR of 19.94 (95% CI, 1.51–263.25). The details of the multivariate Cox regression analysis for PFS are shown in Table 4. Due to the small sample size and heterogeneity of treatment used in the group of patients with PCM-L, the analysis of influence of potential prognostic factors on PFS duration was not performed.

## 4. Discussion

The manuscript summarises the single-centre experience with the diagnosing and treating of PCM of the larynx, an unusual localisation of the disease. Due to the rarity of this condition, our study was extended to include a retrospective analysis of cases reported in the world literature. Both relatively low incidence of PCM itself and, more importantly, the specific localisation of PCM within the larynx contribute to the rarity of this clinical situation, which can be found in approximately 13% of all patients with extramedullary upper aero-digestive tract involvement by extramedullary plasmacytoma [153]. In our patients’ cohort, laryngeal involvement by PCM constituted 0.88% of all malignant larynx tumours and 27.3% among non-epithelial tumours. The diagnosis of sEMP-L was the least frequent, with the relative frequency established at 0.29%. We believe that these are important epidemiological data, considering that the analysis covered a relatively long period from 2009 to 2021 and a significant group of patients with laryngeal neoplasms.

In the analysed cohorts, we were able to identify patients with newly diagnosed sEMP-L, as well as patients with PCM-L. Patients with PCM-L prevailed, which is an expected phenomenon. However, the timing of the diagnosis in our cohort is worth noting. Most of the patients were newly diagnosed. It is an interesting observation, taking into consideration, that the incidence of any extramedullary disease in the relapsed/refractory setting of PCM is higher than in newly diagnosed patients, as reviewed in [154]. Additionally, most of the patients were referred to the otorhinolaryngology department first before the haematology consultation. Therefore, it may be hypothesized that a proportion of PCM patients with laryngeal involvement may remain not diagnosed. The potential reasons for the low referral rate for otorhinolaryngology consultations in patients with some laryngeal symptoms and advanced relapsed/refractory PCM may include the patient’s poor general condition with other important symptoms including significant bone pain or renal failure as well as subsiding of laryngeal symptoms once the systemic treatment is initiated.

The patients mainly presented with unspecific symptoms, typical for all laryngeal tumours, i.e., dyspnoea, hoarseness, and cough (Table 1, Table 2 and Table 3). The symptoms reported in the literature additionally comprised dysphonia, dysphagia, stridor, sore throat, foreign body sensation in the throat, or intermittent wheezing, depending on the involved larynx structures [113,116,128]. Of note, none of the reported symptoms is pathognomonic for PCM of the larynx. Nevertheless, the occurrence of any of these symptoms should render the diagnostic workup. For extramedullary plasmacytomas, ESMO guidelines recommend either whole-body magnetic resonance imaging (MRI) or a positron emission tomography/computed tomography (PET/CT) ([155]. Although not supported by the guidelines, it seems reasonable to additionally incorporate image-enhanced endoscopy techniques in cases of a suspected laryngeal involvement, which are widely used in other laryngeal disorders [156,157].

Males prevailed in the analyzed cohort alike in the published cases as depicted in Table 2 and Table 3, where they constituted 66.4% and 79.5% of all patients with sEMP-L and PCM-L, respectively. Patients with sEMP-L were significantly younger than patients with PCM-L—a median of 65 years and 81 years in the group of our patients and 56 and 64 in the patients from the literature. Even though the age ratio was similar in our literature series, the numeric difference in age was striking, which may point to the fact that either we had missed a substantial proportion of younger patients or, on the contrary, that elderly patients were not qualified for otorhinolaryngology workup by others. Such a phenomenon is known from other hematologic malignancies, e.g., myelodysplastic syndromes or lymphomas, where elderly patients do not undergo all recommended diagnostic procedures. Cartilage involvement was significantly more frequent in patients with laryngeal involvement in the course of systemic disease (30.8% vs. 2.2%).

The treatment differed among patients with sEMP-L and patients with PCM-L. In the case of sEMP-L, the patients from both our group and the literature group were qualified for local treatment either with high-dose radiotherapy, in line with ESMO recommendation [155] or surgery with consolidative radiotherapy or surgery alone. No outcome differences were noted between any of these treatment strategies. Furthermore, we did not observe any significant impact on PFS of the dose of radiotherapy (≤4000 cGy vs. >4000 cGy). However, this result should be treated with caution as the group treated with lower doses was small. Interestingly, most of the patients with PCM-L from our cohort received solely palliative treatment. In the literature group, the patients were treated with a variety of different protocols (Table 3), depending on the period covered by the report, including—in the newer studies—proteasome inhibitors or immunomodulatory agents. Three out of thirty-five patients (8.6%) underwent autologous hematopoietic cell transplantation. We did not identify any patients treated with monoclonal antibodies, e.g., daratumumab or CAR-T (chimeric antigen receptor T-cells) for PCM-L. However, based on reports where these treatment modalities were used for extramedullary disease, e.g., [158,159] it may be anticipated that they would also be effective in laryngeal involvement.

The prognosis of patients with newly diagnosed sEMP-L in our cohort was good, and no patient experienced progression once a response was obtained during the follow-up period. This observation is in line with the reports from the literature [9,10,15,16]. The median OS for all patients was not reached during the observation time, while the 5-year estimate was established at 86% (95% CI, 79–94%), and the 10-year OS at 81% (72–90%). Nevertheless, it should be kept in mind that both local recurrence or progression to systemic PCM [153], as well as the development of treatment-related myeloid neoplasms, i.e., myelodysplastic syndrome (t-MDS) or acute myeloid leukaemia (t-AML), are possible. Therefore, careful surveillance over the patients is necessary. Contrary to sEMP-L, the prognosis of the patients with PCM-L was dismal, both in the case of a new diagnosis and in a relapsed/refractory setting. Most patients did succumb to PCM, with the estimated 5-year OS of 55% (95% CI, 40–74%), equaling the 10-year OS. The laryngeal involvement was not directly associated with the death of the affected patients. The poor outcomes observed in our study and the described literature cases are also in line with other reports, where the extramedullary disease is generally considered a poor prognostic factor [160,161].

Univariate analysis of factors prognostic for PFS in sEMP-L based upon all patients identified male sex and cartilage involvement as factors negatively affecting the outcome. In multivariate analysis, only the cartilage involvement retained its statistical significance, with an HR of 19.94 (95% CI, 1.51–263.25). The explanation of worse prognosis in patients with cartilage involvement is not known. However, it may potentially include differences in tumour biology such as plasmacytoma arising in the area of osseous metaplasia of the cartilage [145], crossing of the mechanical barrier that facilitates extralaryngeal spread or altered radiosensitivity. Based upon this observation, it may be hypothesized that patients with sEMP-L with cartilage involvement should not be treated only with local therapies but also with consolidative systemic treatment. This hypothesis, however, requires verification in future studies, as the group of patients with cartilage involvement was very small.

Factors predisposing to laryngeal involvement by PCM have not been identified. It is, however, worth noting, that in two out of six patients from our retrospective analysis, repeated larynx biopsies revealed chronic inflammation in histopathological examination and only a subsequent biopsy material allowed for a diagnosis of extramedullary disease [8]. It cannot be excluded that chronic inflammation did trigger mutagenesis and, in consequence, the development of PCM at the site of inflammation. This is a well-known phenomenon for other mature B-cell lymphomas [162,163,164,165]. It would be interesting to verify which factors were responsible for the development of chronic inflammation of the larynx. Based on the aetiology of non-Hodgkin’s lymphomas, it is rather unlikely that tobacco and alcohol consumption, the two most important etiological factors for developing laryngeal dysplasia and laryngeal SCC [166] were the ones. The role of human papillomaviruses, affecting solely the epithelial cells, is also unlikely.

It is a well-known fact that the tumour microenvironment (TME) plays an essential role in the pathogenesis of PCM [167,168]. It is advocated that—to allow extramedullary growth—a clone or a subclone must differentiate to thrive and grow independently of the specific bone marrow microenvironment. Apart from the tumour intrinsic factors, the anti-PCM drugs may also influence the interplay between TME and PCM cells and either slow down the immune escape of PCM cells and disease progression or, on the contrary, affect the immune surveillance to facilitate the processes. In the case of bortezomib, the first-in-class proteasome inhibitor, it was shown that a decrease in chemokine receptor CXCR4 expression caused by the drug might lead to the development of the extramedullary disease [169,170]. Although not analysed in our report due to the unavailability of sufficient data, it would be interesting to see whether some treatment protocols or specific drugs used were associated with the development of secondary laryngeal involvement in the relapsed/refractory setting. It would also be compelling to analyse the specific TME of the laryngeal PCM, both in sEMP-L and PCM-L.

The study’s limitations are its retrospective nature and small sample size. The rarity of the diagnosis precludes, however, the possibility of gathering a large group of patients. The review of the literature data allows to overcome this obstacle to some extent; nevertheless, it may be biased by the lack of including all consecutive patients by the reporting centres. The other vital limitations include the lack of information on the cumulative incidence of laryngeal involvement in the course of PCM, as well as the lack of PCM-specific data, including cytogenetics, the presence of other extramedullary sites, and treatment details. Nevertheless, we believe the study provides important information on extramedullary larynx involvement in PCM. It also indicates that appropriate diagnosis and handling of patients with laryngeal involvement by PCM may be challenging and points to the fact that appropriate education aiming to increase the physicians’ awareness about this phenomenon is necessary to aid the proper diagnostic workup and treatment of the patients.

## 5. Conclusions

In conclusion, we confirm that PCM with laryngeal involvement is sporadic and constitutes less than 1% of malignant laryngeal tumours. It may occur either as a solitary extramedullary neoplasm or accompany the systemic disease with bone marrow involvement, the latter being more common and associated with worse outcomes. The involvement of thyroid or cricoid cartilage adversely influences the outcome of solitary extramedullary plasmacytoma of the larynx.

## Figures and Tables

**Table 1:** Summary of the demographics and clinical data for patients with laryngeal involvement with plasma cell myeloma identified in the retrospective analysis.

Patient	Age (Years)	Sex	Type of Disease	Localisation	Symptoms	Surgery	RTX Dose (cGy)	CTH	PFS from Larynx Involvement (mo)	Survival Form Larynx Involvement (mo)	Death	OS (mo)
Patient 1	70	M	sEMPL	1	1, 2, 3	0	5000	0	18	18	0	NA
Patient 2	59	M	sEMPL	1	0	0	4000	0	111	111	0	NA
Patient 3	72	F	PCM-L	5	1, 3	0	N	1	0.75	0.75	1	42
Patient 4	84	M	PCM-L	1	4	0	4000	1	14	14	1	33
Patient 5	78	M	PCM-L	5	3	0	N	0	1.5	1.5	1	1.5
Patient 6	89	M	PCM-L	4	3	0	N	0	2	2	1	2

sEMPL—solitary extramedullary plasmacytoma of the larynx; PCM-L—plasma cell myeloma with bone marrow and laryngeal involvement, NA—not applicable data; mo—months; M—male; F—female; RTX—radiotherapy; CTH—chemotherapy; PFS—progression-free survival; OS—overall survival; Localisation: 1—supraglottic, 4—supraglottic + glottic, 5—supraglottic + glottic + subglottic; Symptoms: 0—no symptoms, 1—hoarseness, 2—cough, 3—dyspnea, 4—localised pain; RTX: N—no radiotherapy; PFS-Larynx—PFS counted from laryngeal involvement; OS-Larynx—OS counted from laryngeal involvement; Surgery/CTH/Death: 1—yes, 0—no.

**Table 2:** Summary of the demographics and clinical data for patients with solitary plasmacytoma of the larynx identified in the literature review.

No.	Author (Year)	Age (y)	Sex	Localisation	Symptoms	Surgery	RTX Dose (cGy)	CTH	Relapse in the Larynx	Progression to Systemic PCM	Death	PFS (mo)	OS (mo)	Ref.
1	Lu and Zhang (2020)	57	F	1	1	1	5000	0	0	0	0	8	8	[9]
2	Tanrivermis Sayit et al. (2020)	74	F	4	1, 7	0	Y	1	0	0	0	3	3	[10]
3	Ong et al. (2020)	55	M	1	NA	NA	NA	NA	NA	NA	NA	NA	NA	[11]
4	Brandt et al. (2020)	54	F	1	1, 2	0	5000	0	0	0	0	18	18	[4]
5	Ge et al. (2018)	46	M	1	3, 6	0	5500	1	0	0	0	60	60	[3]
6	Krebs et al. (2017)	77	M	3	4, 5	0	4600	0	0	0	0	26	26	[12]
7	Wang et al. (2015)	43	M	6	1, 2, 4	1	N	0	1	1	0	12	60	[13]
8	Haser et al. (2015)	72	M	5	1, 4, 8	0	Y	0	0	0	0	12	12	[14]
9	Pino et al. (2015)	65	M	1	1, 2, 7	1	4600	0	0	0	0	54	54	[15]
10	Xing et al. (2015)	47	F	4	1	1	5000	0	0	0	0	18	18	[16]
11	Abrari et al. (2014)	56	M	2	4,10	0	Y	0	0	0	0	NA	NA	[17]
12	Loyo et al. (2013)	80	F	4	1, 2, 4, 8	1	N	0	0	0	0	NA	NA	[18]
13	Ghatak et al. (2013)	29	F	4	1, 4, 7	0	5000	0	0	0	0	16	16	[19]
14	Ramírez-Anguiano et al. (2012)	57	M	5	2, 4, 8	0	5400	0	0	0	0	18	18	[20]
15	Pinto et al. (2012)	49	F	1	2	1	N	0	0	0	0	NA	NA	[21]
16	Kim et al. (2012)	58	M	1	0	1	N	0	0	0	0	24	24	[22]
17	Ravo et al. (2012)	56	M	1	1, 4, 7	1	5000	0	0	0	0	5	5	[23]
18	De Zoysa et al. (2012)	62	F	2	2	0	Y	0	0	0	0	2	2	[24]
19	Pichi et al. (2011)	73	M	5	1, 4	0	4000	0	0	1	1	12	24	[25]
20	Zhang et al. (2010)	56	F	1	1, 2	1	N	0	0	0	0	24	24	[26]
21	González Guijarro et al. (2010)	11	M	4	2	1	4500	0	0	0	0	36	36	[27]
22	Pratibha et al. (2009)	49	M	6	1	0	4400	0	0	0	0	6	6	[28]
23	Fernández-Aceñero et al. (2009)	42	F	1	2, 7	0	Y	0	0	0	0	NA	NA	[29]
24	Iseri et al. (2009)	46	F	1	NA	1	Y	1	0	0	0	24	24	[30]
25	Bilić et al. (2009)	64	M	1	NA	0	N	1	NA	NA	NA	21	21	[31]
26	Rutherford et al. (2009)	13	F	3	NA	1	Y	0	0	0	NA	1	1	[32]
27	Vanan et al. (2009)	16	F	2	1	0	5040	0	0	0	0	12	12	[33]
28	Straetmans and Stokroos (2008)	57	M	1	NA	1	5000	0	1	0	0	2	24	[34]
29	Ozbilean Acar et al. (2008)	43	F	2	NA	1	N	0	0	0	0	24	24	[35]
30	Lewis et al. (2007)	71	M	1	1	1	Y	0	0	0	0	24	24	[36]
31	Kusunoki et al. (2007)	76	F	1	0	0	N	0	0	0	0	6	6	[37]
32	Velez et al. (2007)	64	M	4	1	1	Y	0	0	0	0	36	36	[38]
33	Nakashima et al. (2006)	39	M	1	0	1	6000	0	0	0	0	72	72	[39]
34	Nakashima et al. (2006)	59	M	1	0	1	N	0	0	0	0	180	180	[39]
35	Mackiewicz-Nartowicz et al. (2005)	79	F	2	1	1	N	0	0	0	0	NA	NA	[40]
36	Coskun et al. (2005)	NA	NA	NA	NA	0	Y	0	0	NA	0	NA	NA	[41]
37	Chao et al. (2005)	60	M	1	5, 1	0	5000	0	0	0	1	37	37	[42]
38	Sakiyama et al. (2005)	47	F	3	0	0	5000	1	1	0	0	4	88	[43]
39	Yavas et al. (2004)	43	F	2	1	0	N	0	0	0	0	NA	NA	[44]
40	Michalaki et al. (2003)	46	F	NA	NA	0	4500	0	0	0	0	49	49	[45]
41	Michalaki et al. (2003)	59	M	NA	NA	0	4000	0	0	0	0	67	67	[45]
42	Kamijo et al. (2002)	84	M	1	1	1	6000	0	0	0	0	24	24	[46]
43	Takeda et al. (2002)	53	F	3	NA	0	4600	0	NA	NA	NA	NA	NA	[47]
44	Soni et al. (2002)	65	M	3	1, 3, 4, 7, 8	0	6000	0	0	0	0	25	25	[48]
45	Strojan et al. (2002)	65	M	1	NA	0	6000	0	0	0	1	93	93	[49]
46	Strojan et al. (2002)	72	M	2	NA	0	4600	0	0	0	1	56	56	[49]
47	Strojan et al. (2002)	50	F	1	NA	0	5000	0	0	0	0	27	27	[49]
48	Maheshwari et al. (2001)	65	M	3	3,4	0	Y	0	0	0	0	12	12	[50]
49	Nagasaka et al. (2001)	12	F	3	1,4	1	4320	0	0	0	0	48	48	[51]
50	Abe et al. (2001)	62	F	NA	NA	1	6500	0	NA	NA	NA	NA	NA	[52]
51	Rakover et al. (2000)	38	M	2	1, 5	1	5000	0	1	0	0	5	46	[53]
52	Rosenblatt et al. (1999)	40	M	2	1	0	5000	0	0	0	0	47	47	[54]
53	Furukido et al. (1999)	78	F	2	NA	1	N	0	NA	NA	NA	NA	NA	[55]
54	Yusumatsu et al. (1999)	77	M	1	NA	1	6000	0	NA	NA	NA	NA	NA	[56]
55	Alexiou et al. (1999)	69	M	NA	NA	1	N	0	0	0	0	62	62	[57]
56	Alexiou et al. (1999)	40	M	1	NA	1	Y	0	0	0	0	20	20	[57]
57	Hotz et al. (1999)	63	M	NA	4	1	4000	0	0	0	0	108	108	[58]
58	Hotz et al. (1999)	45	M	NA	7	1	5500	0	NA	0	0	108	108	[58]
59	Nowak-Sadzikowska and Weiss (1998)	34	M	1	1	0	5400	0	0	0	0	120	120	[59]
60	Nowak-Sadzikowska and Weiss (1998)	50	M	2	1	0	6000	0	0	0	0	120	120	[59]
61	Nowak-Sadzikowska and Weiss (1998)	36	M	1	0	0	5400	0	0	0	0	120	120	[59]
62	Nowak-Sadzikowska and Weiss (1998)	68	F	1	1	0	5400	0	0	0	0	120	120	[59]
63	Nowak-Sadzikowska and Weiss (1998)	48	M	2	1	0	6000	0	0	0	0	120	120	[59]
64	Weiss et al. (1998)	34	M	1	1	0	5400	0	0	0	0	120	120	[60]
65	Weiss et al. (1998)	50	M	2	1	0	5400	0	0	0	0	120	120	[60]
66	Weiss et al. (1998)	36	M	1	0	0	6000	0	0	0	0	120	120	[60]
67	Weiss et al. (1998)	68	F	1	1	0	5400	0	0	0	0	120	120	[60]
68	Welsh et al. (1998)	59	M	1	3,4	0	5000	0	0	0	0	42	42	[61]
69	Bhattacharya et al. (1998)	49	F	1	7,10	0	NA	NA	0	0	1	3	3	[62]
70	Sulzner et al. (1998)	49	M	1	3	0	4500	0	0	0	0	61	61	[63]
71	Susnerwala et al. (1997)	79	F	NA	NA	0	3500	0	0	0	0	132	132	[64]
72	Susnerwala et al. (1997)	65	M	NA	NA	0	4500	0	0	0	0	52	52	[64]
73	Han et al. (1996)	34	M	2	NA	1	5000	0	NA	NA	NA	NA	NA	[65]
74	Rodriguez-de-Velasquez et al. (1996)	33	F	5	2	NA	NA	NA	NA	NA	NA	NA	NA	[66]
75	Abe et al. (1995)	53	F	1	NA	0	4500	0	NA	NA	NA	NA	NA	[67]
76	Narożny et al. (1995)	51	M	1	1, 7, 10	0	6000	0	0	1	1	12	12	[68]
77	Narożny et al. (1995)	60	M	1	2, 4	1	5400	0	0	0	0	120	120	[68]
78	Narożny et al. (1995)	55	F	1	7	1	N	0	0	0	0	12	12	[68]
79	Narożny et al. (1995)	57	M	2	1	1	N	0	0	0	0	12	12	[68]
80	Zbären and Zimmermann (1995)	88	M	1	1, 3	1	N	0	0	0	0	72	72	[69]
81	Zbären and Zimmermann (1995)	71	M	1	1	1	N	0	0	0	0	12	12	[69]
82	Rolins et al. (1995)	43	M	1	0	0	N	0	0	0	0	36	36	[70]
83	Mochimatsu et al. (1993)	54	M	1	NA	1	5000	0	NA	1	0	144	144	[71]
84	Weissmann et al. (1993)	76	M	5	1, 3, 10	1	Y	0	0	0	0	NA	NA	[72]
85	Jankowska-Kuc et al. (1993)	58	M	4	1, 7	1	5950	0	0	0	0	36	36	[73]
86	Barbu et al. (1992)	69	M	1	2, 7	0	Y	0	0	0	0	36	36	[74]
87	Tateno et al. (1990)	54	M	1	NA	1	N	0	NA	NA	NA	NA	NA	[75]
88	Kost (1990)	43	M	6	1	1	7000	0	0	0	NA	NA	84	[76]
89	Agatsuma et al. (1989)	74	M	6	NA	1	4000	0	NA	NA	NA	NA	NA	[77]
90	Gambino (1988)	47	M	1	5, 7	1	Y	0	NA	NA	0	NA	NA	[78]
91	Gaffney et al. (1987)	80	M	NA	NA	0	4159	0	0	0	0	7	7	[79]
92	Gadomski et al. (1986)	54	M	2	1, 6, 7	1	N	1	0	NA	1	180	180	[80]
93	Gadomski et al. (1986)	51	M	1	1, 6, 7	0	4500	0	0	NA	0	60	60	[80]
94	Burke et al. (1986)	53	M	1	1, 3, 4	0	N	1	0	0	0	10	10	[81]
95	Gormley et al. (1985)	78	F	3	1, 4, 8	1	3250	0	0	0	0	NA	NA	[82]
96	Maniglia and Xue (1983)	64	F	5	1, 4	1	5000	0	0	0	1	12	12	[83]
97	Ferlito et al. (1982)	45	M	1	1	0	N	1	0	1	0	29	74	[84]
98	Bjelkenkrantz et al. (1981)	NA	NA	1	NA	1	5000	0	0	0	0	84	84	[85]
99	Bush et al. (1981)	52	F	1	NA	0	5500	0	0	0	1	36	36	[86]
100	Bush et al. (1981)	34	F	NA	NA	1	5500	0	0	0	0	42	42	[86]
101	Endo et al. (1979)	68	M	3	NA	0	N	1	NA	NA	NA	NA	NA	[87]
102	Singh et al. (1979)	42	F	1	NA	1	Y	0	NA	0	NA	NA	29	[88]
103	Woodruff et al. (1979)	64	F	1	NA	0	Y	0	0	0	1	72	72	[89]
104	Woodruff et al. (1979)	34	F	1	NA	0	Y	0	0	0	0	NA	NA	[89]
105	Cohen et al. (1978)	15	F	3	4	1	4000	0	1	0	0	3	22	[90]
106	Pahor (1978)	62	M	6	1, 4, 7	1	4500	1	1	0	0	21	31	[91]
107	Gorenstein et al. (1977)	58	M	4	1	1	Y	0	NA	0	0	36	36	[92]
108	Gorenstein et al. (1977)	63	M	4	1	1	Y	0	NA	0	0	300	300	[92]
109	Gorenstein et al. (1977)	59	M	3	4, 8	1	N	0	NA	0	1	60	60	[92]
110	Gorenstein et al. (1977)	42	M	2	1	1	N	0	NA	0	0	60	60	[92]
111	Gorenstein et al. (1977)	61	M	4	1, 7	0	Y	0	NA	0	0	72	72	[92]
112	Petrovich et al. (1977)	74	M	1	10	0	7312	0	0	0	0	24	88	[93]
113	Muller and Fischer (1976)	44	M	1	1, 8	NA	NA	NA	NA	NA	NA	NA	NA	[94]
114	Horiuchi et al. (1973)	51	F	1	NA	1	N	0	NA	NA	NA	NA	NA	[95]
115	Booth et al. (1973)	54	F	4	NA	1	N	0	1	0	1	NA	264	[96]
116	Stone and Cole (1971)	67	M	1	NA	0	Y	1	0	0	0	10	10	[97]
117	Touma (1971)	65	M	4	NA	1	N	0	0	0	0	NA	NA	[98]
118	Kakar et al. (1970)	45	F	1	1, 4	1	N	0	0	0	0	13	13	[99]
119	Poole and Marchetta (1968)	41	M	NA	NA	1	Y	0	NA	0	1	NA	41	[100]
120	Nabar (1968)	60	F	1	NA	1	N	0	0	0	0	132	132	[101]
121	Nabar (1968)	55	M	1	NA	0	Y	0	0	0	1	NA	NA	[101]
122	Nabar (1968)	46	F	1	NA	1	N	0	0	0	0	NA	NA	[101]
123	Kraska (1967)	40	F	1	1, 4	0	7290	0	0	0	0	14	14	[102]
124	Studencki (1966)	54	F	4	1, 4, 10	0	5500	0	0	0	0	7	7	[103]
125	Todd (1965)	65	M	1	NA	0	2500	0	1	1	1	60	96	[104]
126	Todd (1965)	43	M	1	NA	0	4800	0	0	0	0	60	60	[104]
127	Webb et al. (1962)	55	F	4	1	1	N	0	0	0	0	132	132	[105]
128	Webb et al. (1962)	32	M	3	1	1	Y	0	0	0	0	120	120	[105]
129	Webb et al. (1962)	46	M	1	1, 9	1	Y	0	1	1	1	168	330	[105]
130	Krotz and Ritterhoff (1961)	75	M	NA	NA	0	Y	0	1	NA	1	NA	NA	[106]
131	Clark (1957)	60	F	1	1, 4, 7	1	N	0	0	0	0	1	1	[107]
132	Carson et al. (1955)	52	M	1	NA	1	Y	0	0	1	0	54	54	[108]
133	Carson et al. (1955)	73	M	1	NA	1	N	0	NA	NA	1	156	156	[108]
134	Korkis (1954)	42	F	1	NA	1	N	0	0	0	0	NA	NA	[109]
135	Priest (1952)	50	M	4	2, 10	1	N	0	1	0	0	9	48	[110]
136	Ewing and Foote (1952)	76	M	1	1	NA	Y	NA	0	0	0	6	6	[111]
137	Costen (1951)	52	M	1	NA	0	Y	0	0	1	0	NA	12	[112]
138	Mattick (1950)	41	M	4	1, 3, 4, 7, 10	1	N	0	1	1	1	27	41	[113]
139	Rawson et al. (1950)	59	F	1	NA	1	Y	0	0	1	0	36	NA	[114]
140	Stout and Kenney (1949)	46	M	1	4, 6	1	8400	0	1	0	0	3	171	[115]
141	Hodge and Wilson (1948)	53	M	4	1, 3, 5, 10	1	N	0	0	0	0	12	12	[116]
142	Lumb and Prossor (1948)	34	M	3	NA	0	Y	0	1	0	0	24	30	[117]
143	Lumb and Prossor (1948)	20	M	7	NA	1	Y	0	0	0	0	90	90	[117]
144	Clerf (1946)	75	M	1	NA	1	N	0	0	0	1	13	13	[118]
145	Jaeger (1942)	67	F	NA	NA	0	Y	0	0	0	1	6	6	[119]
146	Haven and Parkhill (1941)	62	M	1	NA	1	Y	0	0	0	0	54	54	[120]
147	Ringertz (1938)	59	M	2	1	0	Y	0	0	0	0	48	48	[121]
148	Ringertz (1938)	73	M	1	NA	1	N	0	0	0	0	12	12	[121]
149	Ringertz (1938)	57	F	1	NA	1	Y	0	0	0	1	48	48	[121]
150	Heindl (1933)	NA	NA	NA	NA	1	N	0	0	0	0	36	NA	[122]
151	Blacklock and Macartney (1932)	64	M	NA	NA	1	N	0	0	0	0	NA	24	[123]
152	Wachter (1914)	48	F	NA	NA	1	N	0	0	0	0	5	142	[124]

NA—non-available data; y—years (age); mo—months; M—male; F—female; RTX—radiotherapy; CTH—chemotherapy; PCM—plasma cell myeloma; PFS—progression-free survival; OS—overall survival; Ref.—references; Localisation: 1—supraglottic, 2—glottic, 3—subglottic, 4—supraglottic + glottic, 5—glottic + subglottic, 6—supraglottic + glottic + subglottic, 7—supraglottic + subglottic; Symptoms: 0—no symptoms, 1—hoarseness, 2—dysphonia, 3—cough, 4—dyspnea, 5—globus sensation, 6—sore throat, 7—dysphagia, 8—stridor, 9—odynophagia, 10 —other; RTX: Y—non-available dose, N—no radiotherapy, NA—no information; Surgery/CTH/Local relapse/Progression to MM/Death: 1—yes, 0—no.

**Table 3:** Summary of the demographics and clinical data for patients with secondary laryngeal involvement during systemic plasma cell myeloma with bone marrow involvement identified in the literature review.

No.	Author (Year)	Age (y)	Sex	Localisation in the Larynx	Symptoms at Larynx Involvement	Involvement of Larynx at PCM Diagnosis	Surgery	RTX Dose (cGy)	CTH	PFS (mo)	OS (mo)	Death	Ref.
1	You and Bhuta (2019)	68	M	4	2, 5	1	0	N	1	132	146	1	[6]
2	Doğan et al. (2019)	44	M	3	4	NA	0	N	1	NA	NA	NA	[7]
3	Doğan et al. (2019)	55	M	6	1, 4	NA	0	N	1	NA	NA	NA	[7]
4	Doğan et al. (2019)	70	M	3	4, 6	NA	0	N	1	NA	NA	NA	[7]
5	Steinke et al. (2019)	81	M	4	2, 4, 7	1	0	6000	0	NA	NA	0	[125]
6	Allegra et al. (2017)	68	M	4	2, 4, 6	0	0	N	1	6	6	0	[126]
7	Nochikattil et al. (2016)	44	F	1	1, 7	0	NA	NA	NA	NA	NA	0	[127]
8	Gan et al. (2014)	55	M	3	2, 4, 7, 10	0	1	N	1	24	24	0	[128]
9	Mitchell et al. (2013)	63	M	4	1, 5	1	0	Y	1	36	NA	0	[129]
10	Alherabi et al. (2013)	77	M	1	6	0	NA	NA	0	NA	NA	0	[130]
11	Grobman et al. (2012)	58	M	1	2	1	0	3000	0	120	124	0	[131]
12	Kumar et al. (2011)	63	M	2	1, 4, 6, 9	1	1	N	1	48	49	1	[132]
13	Straetmans and Stokroos (2008)	62	F	3	7	1	0	N	1	36	48	0	[34]
14	Oztürk et al. (2008)	65	M	3	4	1	0	NA	1	NA	NA	0	[133]
15	Dispenza et al. (2007)	69	F	4	1, 6	0	0	N	1	12	12	0	[134]
16	Shimada et al. (2006)	72	M	NA	0	0	1	5000	0	36	36	0	[135]
17	Nampoothiri et al. (2006)	65	M	3	7	0	0	N	1	NA	NA	0	[136]
18	Luppino et al. (2005)	NA	NA	2	1, 6	1	NA	NA	NA	NA	NA	NA	[137]
19	Gross et al. (2002)	50	M	4	1, 4, 5, 7	0	0	4500	1	3	3	1	[138]
20	Sosna et al. (2002)	54	M	4	1, 4, 10	0	0	3600	1	NA	NA	1	[139]
21	Aslan et al. (2001)	70	M	3	1, 10	0	0	5000	0	6	6	0	[140]
22	Saad et al. (2001)	79	M	6	8	1	0	N	1	30	36	1	[141]
23	Uppal et al. (2001)	54	M	4	7,10	1	0	Y	1	36	37	1	[142]
24	Nofsinger et al. (1997)	72	M	3	1, 4	1	0	6300	1	6	NA	1	[143]
25	Gabryś et al. (1996)	63	M	3	NA	1	0	Y	1	40	40	1	[144]
26	Van Dyke et al. (1996)	62	M	4	1, 10	1	0	4000	1	12	54	0	[145]
27	Rabinov et al. (1993)	48	M	3	1, 7	1	1	Y	0	NA	NA	0	[146]
28	Werner et al. (1991)	48	F	3	NA	0	0	N	1	14	14	1	[147]
29	Georghiou and Hogg (1988)	71	M	3	1, 3, 7, 10	0	0	Y	1	1	1	1	[148]
30	Jones et al. (1988)	73	M	6	4, 6, 7	1	0	N	1	18	19	1	[149]
31	Maniglia and Xue (1983)	61	F	1	1, 3, 4	0	0	Y	0	2	38	1	[83]
32	Maniglia and Xue (1983)	73	M	5	1, 4	0	0	5600	0	NA	NA	0	[83]
33	East (1978)	47	F	1	1, 2, 3, 6	1	0	3600	1	14	17	1	[150]
34	Pirkey (1957)	49	M	3	1, 3, 4, 7, 10	0	0	Y	0	3	6	1	[151]
35	Shaw (1972)	56	M	1	NA	NA	1	6500	1	NA	36	1	[152]

PCM—plasma cell myeloma; NA—non-available data; y—years (age); mo—months; M—male; F—female; RTX—radiotherapy; CTH—chemotherapy; PFS—progression-free survival; OS—overall survival; Ref.—references; Localisation: 1—supraglottic, 2—glottic, 3—subglottic, 4—supraglottic + glottic, 5—glottic + subglottic, 6—supraglottic + glottic + subglottic, Symptoms: 0—no symptoms, 1—hoarseness, 2—dysphonia, 3—cough, 4—dyspnea, 5—globus sensation, 6—dysphagia, 7—stridor, 8—odynophagia, 9—localised pain, 10—other; RTX: Y—non-available dose, N—no radiotherapy, NA—no information; Surgery/CTH/Death: 1—yes, 0—no.

**Table 4:** Results of multivariate Cox model with backward selection analysis of factors with potential influence on progression-free survival in patients with solitary extramedullary plasmacytoma of the larynx (sEMPL).

Parameter	Hazard Ratio	95% Confidence Interval	*p*
Involvement of cartilage	19.51	1.46–259.66	0.024
Male gender	8.00	0.88–72.55	0.065
Radiotherapy	0.14	0.01–1.52	0.106
Surgery	0.23	0.02–2.66	0.237
Combined surgery with radiotherapy	0.24	0.02–242	0.227

## Data Availability

Not applicable.
